# The Eminent Role of microRNAs in the Pathogenesis of Alzheimer's Disease

**DOI:** 10.3389/fnagi.2021.641080

**Published:** 2021-03-15

**Authors:** Mohammad Samadian, Mahdi Gholipour, Mohammadreza Hajiesmaeili, Mohammad Taheri, Soudeh Ghafouri-Fard

**Affiliations:** ^1^Skull Base Research Center, Loghman Hakim Hospital, Shahid Beheshti University of Medical Sciences, Tehran, Iran; ^2^Department of Medical Genetics, Shahid Beheshti University of Medical Sciences, Tehran, Iran; ^3^Urology and Nephrology Research Center, Shahid Beheshti University of Medical Sciences, Tehran, Iran; ^4^Urogenital Stem Cell Research Center, Shahid Beheshti University of Medical Sciences, Tehran, Iran

**Keywords:** Alzheimer's disease, miRNA, marker, expression, polymorphism

## Abstract

Alzheimer's disease (AD) is an irrevocable neurodegenerative condition characterized by the presence of senile plaques comprising amassed β-amyloid peptides (Aβ) and neurofibrillary tangles mainly comprising extremely phosphorylated Tau proteins. Recent studies have emphasized the role of microRNAs (miRNAs) in the development of AD. A number of miRNAs, namely, miR-200a-3p, miR-195, miR-338-5p, miR-34a-5p, miR-125b-5p, miR-132, miR-384, miR-339-5p, miR-135b, miR-425-5p, and miR-339-5p, have been shown to participate in the development of AD through interacting with BACE1. Other miRNAs might affect the inflammatory responses in the course of AD. Aberrant expression of several miRNAs in the plasma samples of AD subjects has been shown to have the aptitude for differentiation of AD subjects from healthy subjects. Finally, a number of AD-modifying agents affect miRNA profile in cell cultures or animal models. We have performed a comprehensive search and summarized the obtained data about the function of miRNAs in AD in the current review article.

## Introduction

Alzheimer's disease (AD) is an irrevocable neurodegenerative condition with a progressive course, and it is the chief reason for dementia in the elderly (Prince et al., 2013). AD is characterized by pervasive cognitive defects and memory deficits, leading to the dependence of the majority of AD patients on others for their routine activities. From a pathological point of view, AD is defined by the presence of senile plaques comprising amassed β-amyloid peptides (Aβ) and neurofibrillary tangles mainly comprising extremely phosphorylated Tau proteins (Ballard et al., 2011). The most accepted hypotheses for the development of AD are based on these two main pathological events [i.e., Aβ accumulation and Tau accumulation (Wang et al., 2019a)]. The amyloid precursor protein is converted to Aβ through consecutive enzymatic reactions catalyzed by β-secretase (BACE1) and γ-secretase (containing presenilin 1 and presenilin 2) (Querfurth and LaFerla, 2010). Recent studies have emphasized the role of microRNAs (miRNAs) in the development of AD (Wang et al., 2019a). These ~22 nucleotide transcripts post-transcriptionally regulate the expression of several target genes through binding with 3' UTR and destructing the target transcript or reducing its translation (O'Brien et al., 2018). Sequence complementarity mainly regulates the miRNA/mRNA interactions leading to the ability of one miRNA to target several genes and the possible regulation of one gene by multiple miRNAs. Therefore, miRNAs are potential means for investigating multifactorial disorders such as AD (Iqbal and Grundke-Iqbal, 2010). A leading investigation in this regard has examined the number of brain-associated miRNAs expressed in the human hippocampus specimens obtained from fetal, adult, and AD patients, revealing misregulation of certain miRNAs in the AD brain and their possible contribution to the pathological processes of this disorder (Lukiw, 2007). Dysregulation of other miRNAs has also been verified in multiple studies, and the underlying mechanisms of their contribution in AD have been identified in some cases. We have performed a comprehensive search and summarized the obtained data about the function of miRNAs in AD in this review article.

## Dysregulated miRNAs in AD

Dysregulation of miRNAs has been demonstrated in human AD subjects or animal models of AD. Moreover, several researchers have induced or suppressed the expression of some miRNAs in the cell/animal models of AD to appraise their function in the progression of AD. In a rat model of AD caused by the administration of Aβ_25−35_ into the brain, downregulation of SOX6 and over-expression of miR-129-5p have shortened the dormant escape period and enhanced the time of crossing platforms, repairing the pathological damage, blocking neuronal apoptosis, and decreasing inflammation. Based on the protective effects of miR-129-5p against nerve damage and inflammation, miR-129-5p has been suggested as a candidate for therapeutic options against AD, as it acts to suppress SOX6 (Zeng et al., 2019). Expression of miR-200a-3p has been shown to be repressed in animal and cell models of AD. miR-200a-3p can suppress cell apoptosis, inactivate Bax/caspase-3 axis, and decrease Aβ_1−42_ and tau phosphorylation in cell experiments. Mechanistically, these effects are mediated through the modulation of translocation of BACE1 and PRKACB. Taken together, the neuroprotective impact of miR-200a-3p is accomplished by inhibition of BACE1 expression and subsequent suppression of Aβ production as well as reduction of PKA expression and Tau phosphorylation (Wang et al., 2019b). miR-455-3p has been shown to bind with 3' UTR of APP gene to decrease its expression and reduce expression of mitochondrial fission proteins (Kumar et al., 2019). Mutant APP cells that show expression of miR-455-3p exhibit upregulation of synaptic genes. Over-expression of miR-455-3p in mutant APP cells reduces the number of mitochondria and increases the size of the mitochondria. Taken together, miR-455-3p controls APP processing and protects against mutant APP-associated mitochondrial dysfunction and synaptic anomalies in AD (Kumar et al., 2019). Expression of miR-455-3p has been shown to be increased in postmortem brain samples, fibroblasts, and plasma samples of patients with AD compared with controls (Kumar et al., 2017; Kumar and Reddy, 2018). As a primary event, expression of miR-409-5p has been decreased in an APP/PS1 double transgenic mice model of AD. Over-expression experiments have shown that this miRNA has a harmful impact on neurite outgrowth, reduces neuron survival, and quickens the progression of Aβ_1−42_-associated pathologic events (Guo et al., 2019). In line with the observed downregulation of miR-409-5p in APP/PS1 AD model, Aβ_1−42_ peptide has been shown to downregulate miR-409-5p levels. A luciferase study has shown that Plek is a target of miR-409-5p (Guo et al., 2019). Ectopic expression of miR-409-5p has induced neurotoxic effects and interferes with neuron survival and differentiation, while Plek upregulation could partly protect the neurite outgrowth from these toxic effects. Taken together, reduction of miR-409-5p expression in the early stages of AD might be a self-protective response to lessen the synaptic injury induced by Aβ (Guo et al., 2019). miR-132 is another downregulated miRNA in AD. Experiments in a rat model of AD have shown upregulation of AChE, iNOS, ROS, MDA, MAPK1, and p-MAPK1 and downregulation of SOD, GSH-Px, and miR-132. Over-expression of miR-132 has reversed these markers demonstrating the role of this miRNA in the suppression of hippocampal iNOS expression and oxidative stress through reduction of MAPK1 levels (Deng et al., 2020). However, expression of this miRNA has been demonstrated to be reduced in neurally-originated plasma exosomes of AD subjects (Cha et al., 2019). Table 1 shows the summary of studies that reported decreased levels of miRNAs in AD.

**Table 1:** Downregulated miRNAs in AD subject, animal models of AD, and related cell lines and their functions in progression of AD.

**microRNA**	**Samples**	**Assessed cell line**	**Gene/protein interaction**	**Signaling pathway**	**Function**	**References**
miR-129-5p	90 male-specific pathogen-free (SPF) Sprague-Dawley (SD) rats	Hippocampal neuron cells of rat	SOX6	-	Its upregulation represses apoptosis and inflammatory reactions and attenuates neural injury by targeting SOX6.	Zeng et al., 2019
miR-200a-3p	Plasma samples from 7 patients with AD and 5 age-matched healthy individual, APP/PS1 mice, SAMP8, and SAMR1 mice	NB-1	BACE1, PRKACB	-	Has neuroprotective effects, suppresses apoptosis, and decreases Aβ production through regulating expression of BACE1 and PRKACB	Wang et al., 2019b
miR-326	APPswe/PS1d E9 double transgenic mouse	-	VAV1	JNK signaling pathway	Its overexpression decreased neuronal apoptosis and Aβ accumulation and elevated viability of neuron cells by targeting VAV1.	He et al., 2020
miR-98	70 Kunming mice	Hippocampal neuronal cells	HEY2	Notch signaling pathway	Represses apoptosis of hippocampal neurons and shows enhanced survival of these cells by targeting HEY2 and inactivating the Notch signaling pathway	Chen et al., 2019
miR-196a	60 male Sprague-Dawley mice	HEK-293T	LRIG3	PI3/Akt pathway	Its upregulation ameliorated cognitive decline, inhibited apoptosis, and increased survival of neurons by targeting LRIG3.	Yang et al., 2019a
miR-195	Postmortem human brain tissues and CSF samples from AD patients and MCI subjects, Human ApoE4^+/+^ or ApoE3^+/+^ knock-in (KI) mice	Mouse primary neuron	synj1	-	Its overexpression alleviated cognitive impairment and decreased Aβ deposition and tau hyper-phosphorylation.	Cao et al., 2020
miR-195	SAMP8 and SAMR1 mice	HEK293, N2a	BACE1	-	Its overexpression reduced Aβ production through targeting BACE1.	Zhu et al., 2012
miR-338-5p	Hippocampal tissue samples from patients with AD and normal subjects, 5XFAD transgenic (TG) mice	-	BACE1	NF-κB signaling pathway	Its overexpression prevented Aβ formation, neuroinflammation, cognitive deficit and impaired learning ability by targeting BACE1.	Qian et al., 2019
miR-338-5p	Male C57BL/6 mice and male APP/PS1 transgenic mice	Primary hippocampal neurons	BCL2L11	-	Its overexpression prevented Aβ deposition, cognitive decline, and reduced apoptosis rate of neurons by targeting BCL2L11.	Li et al., 2020a
miR-133b	Serum samples from 105 AD patients and 98 control individuals	SH-SY5Y	EGFR	-	Its overexpression reduced apoptosis rate and improved cell viability.	Wang et al., 2019c
miR-124	Male APP/PS1 transgenic mice	-	C1ql3	-	Its overexpression increased angiogenesis and lowered the accumulation of Aβ and prevented memory decline and learning impairment.	Zhang et al., 2019
miR-124-3p	-	N2a/APP695swe cells	Caveolin-1	PI3K/Akt/GSK3β pathway	Its upregulation abated Tau hyperphosphorylation and cellular apoptosis by targeting Caveolin-1 and modulation of PI3K/Akt/GSK3β pathway.	Kang et al., 2017
miR-101a	Plasma samples from 46 AD patients 60 healthy individuals, APPswe/ PS1ΔE9 transgenic mice	SH-SY5Y	MAPK1	MAPK pathway	Regulates autophagy through targeting MAPK1 and modulating the MAPK pathway	Xiao et al., 2019
miR-22	Serum samples from 33 patients with AD and 30 healthy volunteers, APP/PS1 double transgenic mice	MG cells	GSDMD	-	Its overexpression suppressed secretion of inflammatory factors and pyroptosis also decreased GSDMD expression.	Han et al., 2020
miR-34a	-	SH-SY5Y	Caspase-2	-	Its upregulation suppressed neurotoxicity induced by Aβ through targeting Caspase-2.	Wang et al., 2019c
miR-34a	APP/PS1 transgenic mice	SH-SY5Y, primary cortical neuronal cells	cyclin D1	-	Regulates apoptosis rate and neuronal cell cycle by targeting cyclin D1	Modi et al., 2016
miR-34a-5p miR-125b-5p	Serum samples from 27 AD patients and 27 age-matched control individuals	N2a, MCN	BACE1	-	Their overexpression ameliorated oxidative stress and apoptosis induced by Aβ through targeting BACE1.	Li et al., 2020b
miR-181a	APP/PS1 transgenic mice and male wild-type C67BL/6J mice	Murine brain pericytes	FOXO1	-	Its overexpression alleviated cognitive decline, reduced accumulation of Aβ, and slowed pericyte loss by targeting FOXO1.	Wu et al., 2019
miR-31	Female AD triple-transgenic mice	HT-22, HEK293, SH-SY5Y	APP	-	Its overexpression reduced Aβ accumulation and alleviated neuropathology of AD and memory impairment.	Barros-Viegas et al., 2020
miR-409-5p	APPswe/PS1ΔE9 double transgenic mice	PC12, Neuro2A, HEK293T	Plek	-	Its overexpression expression aggravated cell survival and differentiation and impaired neurite outgrowth by targeting Plek.	Guo et al., 2019
miR-107	CSF samples from 22 AD patients and 10 healthy controls	SH-SY5Y	FGF7	FGFR2/PI3K/Akt pathway	Its upregulation reduced apoptosis and inflammation also elevated proliferation of SH-SY5Y through regulation of FGF7/FGFR2/PI3K/Akt Pathway induced by Aβ.	Chen et al., 2020a
miR-107	-	hCMEC/D3, NHA, HBVP	Endophilin-1	-	Its overexpression inhibited disruption of the blood–brain barrier induced by Aβ and alleviated impaired function of endothelial cells by targeting Endophilin-1.	Liu et al., 2016a
miR-107 miR-103	Post-mortem brain tissues from 12 AD patients and 12 age- and gender-matched control individuals	SK-N-BE, HEK-293	CDK5R1	-	Can be implicated in AD pathogenesis through regulation of CDK5R1 expression and consequently influencing p53 levels	Moncini et al., 2017
miR-212	Plasma sample from 31 AD patients and 31 control subjects	SH-SY5Y, IMR-32	PDCD4	PI3K/AKT signaling pathway	Reduces neurotoxicity of Aβ by targeting PDCD4 regulation of PI3K/AKT signaling pathway	Chang, 2020
miR-433	Serum samples from 118 AD patients and 62 healthy controls	SH-SY5Y, SK-N-SH	JAK2	-	Its overexpression improved the viability of neurons by targeting JAK2. Its expression is associated with MMSE scores.	Wang and Zhang, 2020
miR-132	70 SPF Sprague-Dawley rats	HEK 293T	MAPK1	MAPK1 signal pathway	Suppresses oxidative stress and alleviated cognitive performance by targeting MAPK1	Deng et al., 2020
miR-132	P301S Tau transgenic mice	Primary cortical and hippocampal neuron cultures	Rbfox1, GSK3β, EP300, Calpain 2	-	Has neuroprotective effects including reduces neurotoxicity of Aβ and improves elongation of neurite and decreases neuronal death through targeting Rbfox1, GSK3β, EP300, and Calpain 2	El Fatimy et al., 2018
miR-132 miR-212	Human post-mortem brain tissues from 10 AD patients and 6 control subjects	Primary human neurons, SH-SY5Y	NOS1	-	Low expression of miR-132 and miR-212 disrupted the balance of S-nitrosylation through modulation of NOS1 expression.	Wang et al., 2017
miR-132 miR-212	Brain tissues from 29 AD patients and 16 controls	PC12, primary neurons	PTEN, FOXO3a, P300	AKT signaling pathway	Regulates survival and apoptosis of neuronal cells through targeting PTEN, FOXO3a, and P300.	Wong et al., 2013
miR-132	Post-mortem brain tissues from AD patients, 3xTg-AD mice lacking the miR-132/212 cluster	Neuro2a, Neuro2a APPSwe/Δ9, HEK293T, HEK293-APPSwe	Sirt1	-	Its deletion was associated with increased Aβ production and the establishment of amyloid plaque.	Hernandez-Rapp et al., 2016
miR-132	Brain tissues from AD patients and normal controls, APPPS1 mice	HEK293-APP^swe^	ITPKB	-	Regulates Aβ formation and TAU phosphorylation through targeting ITPKB and modulation of ERK1/2 and BACE1 activity.	Salta et al., 2016
miR-9-5p	-	HT22	GSK-3β	Nrf2/Keap1 signaling	Its overexpression caused a reduction in the apoptosis rate, oxidative stress, and prevention of mitochondrial malfunction by targeting GSK-3β.	Liu et al., 2020
miR-377	-	SH-SY5Y	CDH13	-	Its upregulation enhanced cell proliferation and prevented occurrence apoptosis by targeting CDH13.	Liu et al., 2018
miR-221	Blood samples from 21 AD patients and 17 controls	SH-SY5Y	ADAM10	-	Can be implicated in AD pathogenesis through regulation of ADAM10 expression	Manzine et al., 2018
miR-186	72 male Sprague–Dawley (SD) rats	Hippocampal neuronal cells	IL2	JAK-STAT signaling pathway	Its upregulation inhibited apoptosis and enhanced cell proliferation through targeting IL2 and regulation of the JAK-STAT signaling pathway.	Wu et al., 2018a
miR-330	14 C57 mice	Primary neuron cells obtained from mice	VAV1	MAPK signaling pathway	Its overexpression reduced oxidative stress, ameliorated mitochondrial dysfunction, and decreased the generation of Aβ by targeting VAV1.	Han et al., 2018
let-7f-5p	C57BL/6J-TgN (APP/PS1) ZLFILAS mice	Bone marrow mesenchymal stem cells	Caspase-3	-	Its overexpression inhibited apoptosis induced by Aβ through targeting caspase-3. It also increased the survival rate of MSCs in mouse brain.	Shu et al., 2018
miR-107	60 male C57 mice	-	-	-	Its overexpression alleviated spatial memory dysfunction, hippocampal long-term potentiation and prevented the elimination of pyramidal neurons induced resulted from neurotoxicity of Aβ.	Shu et al., 2018
miRNA-140-5p	Post mortem brain tissues from 21 AD patients and 22 normal subjects	SHSY5Y, CHP212	ADAM10, SOX2	-	Is implicated in AD pathogenesis through targeting ADAM10 and its transcription factor SOX2	Akhter et al., 2018
miR-384	Serum and CSF samples from 32 MCI patients, 45 AD patients, and 50 control individuals	SH-SY5Y, HEK293	BACE-1, APP	-	Its overexpression decreased the expression of BACE-1 and APP so it can contribute to AD pathogenesis.	Liu et al., 2014b
miR-188-5p	Brain tissues from 5 AD patients and 3 controls, 5XFAD mice	Primary hippocampal neuron cells	-	-	Its overexpression alleviated cognitive dysfunction and memory loss also restored synaptic activity.	Lee et al., 2016
miR-193b	Plasma and CSF samples from AD patients, MCI patients and control subjects, APP/PS1 double-transgenic	SH-SY5Y, HEK293	APP	-	Its upregulation downregulated APP expression so it can be implicated in AD pathogenesis.	Liu et al., 2014a
miR-153	APPswe/PSΔE9 mice	SH-SY5Y, HEK-293T, M17	APP, APLP2	-	Its overexpression downregulated expression APP and APLP2 so can be an important factor in the pathogenesis of AD.	Liang et al., 2012
miR-153	Brain tissues from 15 AD patients and 5 normal controls	HeLa, primary human fetal brain cultures	APP	-	Can be implicated in AD pathogenesis through targeting APP and reducing APP expression	Long et al., 2012
miR-16	SAMP8 mice, SAMR1 mice, and BALb/c mice	Neuroblastoma2a and NIH3T3	APP	-	Its upregulation downregulated the expression of APP and consequently prevented APP accumulation.	Liu et al., 2012
miR-339-5p	Frozen brain tissues from 20 AD patients and 5 controls	HeLa, U373 MG, human primary brain cultures	BACE1	-	Can contribute to AD pathogenesis through targeting BACE1	Long et al., 2014
miR-214-3p	CSF samples from eight patients with sporadic AD and 8 age-matched healthy volunteers, SAMR1 and SAMP8 mice	Primary neurons obtained from SAMP8 mice, SH-SY5Y	Atg12	-	Its upregulation decreased autophagy and apoptosis rate in neuronal cells and improved cognitive function through targeting Atg12.	Zhang et al., 2016a
miR-222	APPswe/PSΔE9 mice	SH-SY5Y, HEK-293T	p27Kip1	-	Regulates cell cycle by targeting p27Kip1 so can be involved in AD pathogenesis	Wang et al., 2015a
miR-29c	CSF samples from 30 AD patients and 30 age-matched controls	Primary hippocampal neurons	DNMT3	-	Regulates neuronal proliferation by targeting DNMT3 and regulation of BDNF expression.	Yang et al., 2015
miR-101	-	Primary hippocampal neurons	APP	-	Its overexpression lead to decreased accumulation of Aβ through targeting APP.	Vilardo et al., 2010
miR-181c	SAMP8 and SAMR1 mice	HT-22, HEK293A	crmp2	-	Can be implicated in the pathogenesis of AD by targeting crmp2 and downregulation of crmp2 expression	Zhou et al., 2016
miR-135b	Blood samples from 25 AD patients and 25 age-matched healthy individuals,	Primary hippocampal cells derived from SAMR1 mice	BACE1	-	Its overexpression elevated cell proliferation and improved memory function and learning capacity by targeting BACE1.	Zhang et al., 2016b

Although several studies have reported downregulation of miR-132 in AD (Wong et al., 2013; El Fatimy et al., 2018; Cha et al., 2019; Deng et al., 2020), Liu et al. have reported high levels of miR-132 in patients with mild cognitive impairment and AD vs. normal individuals. They have shown the impact of miR-132 upregulation in the induction of apoptosis in neurons through increasing Bax/Bcl-2 ratio (Liu and Zhang, 2019). Moreover, they have reported that miR-132 increases Tau phosphorylation and expression levels of Rb, Histone H1, and CDK-5. Collectively, they have suggested that miR-132 participates in AD by controlling cell apoptosis and the GTDC-1/CDK-5/Tau phosphorylation axis (Liu and Zhang, 2019). In addition to GTDC-1, miR-132 is also known to regulate the expression of synaptic proteins *via* complement C1q (Xu et al., 2019). Similarly, expression of miR-132 has been shown to be decreased in AD-derived plasma exosomes (Cha et al., 2019). miR-128 has also been over-expressed in the brain samples of AD patients (Liu et al., 2019). Experiments in AD mice have demonstrated parallel upregulation of miR-128 and downregulation of PPARγ in the cerebral cortex. The interaction between these two transcripts has been validated through functional assays. miR-128 silencing has suppressed AD-like features, amyloid plaque creation, Aβ production, and inflammation in AD mice through upregulating PPARγ (Liu et al., 2019). miR-425-5p is another upregulated miRNA in patients with AD and the cellular model of AD. Upregulation of miR-425-5p has induced cell apoptosis, stimulated expression of GSK-3β, and enhanced tau phosphorylation through targeting HSPB8 (Yuan et al., 2020). miR-146a is also upregulated in AD and participates in the pathogenesis of this condition *via* targeting Lrp2 and inhibiting the Akt signaling pathway, modulating ROCK1 expression and decreasing Tau phosphorylation, and influencing inflammatory responses *via* modulation of IRAK-1 (Cui et al., 2010; Wang et al., 2016). Insulin and liver X receptor (LXR) activators have been shown to increase the miR-7-1 levels. Expression of this miRNA has changed within the brains of diet-induced obese animals as well as AD patients, which is in parallel with the downregulation of its target genes IRS-2 and IDE. Upregulation of miR-7 has enhanced extracellular Aβ levels in neurons and interfered with the eradication of Aβ by microglia. Collectively, insulin can act *via* the HNRNPK-miR-7 cascade to post-transcriptionally affect metabolic pathways in AD (Fernández-de Frutos et al., 2019). Table 2 lists upregulated miRNAs in AD.

**Table 2:** Upregulated miRNAs in AD subject, animal models of AD or related cell lines and their functions in progression of AD.

**microRNA**	**Samples**	**Assessed cell line**	**Gene/protein interaction**	**Signaling pathway**	**Function**	**References**
miR-132	Frozen human postmortem brain specimens from 10 patients with mild cognitive impairment, 10 patients with AD, and 10 controls	Human cortical neuron culture	GTDC-1	-	Enhances neuronal apoptosis and Tau phosphorylation by targeting GTDC-1	Liu and Zhang, 2019
miR-30b	Human hippocampal tissues, C57BL/6J mice, and 5XFAD APP transgenic mice	NG108–15, HEK 293/293T	ephB2, sirt1, GluA2	NF-κB signaling pathway	Disrupts cognitive and synaptic functions and its knockdown reverses this effect by targeting ephB2, sirt1, and GluA2	Song et al., 2019
miR-128	APP/PSA/Tau triple transgenic mice and C57BL/6 mice	N2a cells	PPARγ	-	Its knockout suppressed AD development, Aβ production, and inflammatory reactions by targeting PPARγ.	Liu et al., 2019
miR-128	Plasma samples from 20 patients with AD and age and education-matched normal subjects	MCN, Neuro2a	PPAR-γ	-	Its inhibition abated neurotoxicity of Aβ through regulation of PPAR-γ and deactivated NF-κB.	Geng et al., 2018
miR-7	Postmortem human brains from AD patients and individuals without severe neurological and psychological disorders male C57BL/6 mice	N2a cell, BV-2	INSR, IRS-2, IDE	Insulin signaling	Enhances extracellular Aβ and suppresses its clearance by regulating Insulin signaling through targeting INSR, IRS-2, and IDE	Fernández-de Frutos et al., 2019
miR-592	54 Sprague-Dawley (SD) male rats established as an AD model	Astrocyte culture	KIAA0319	Keap1/Nrf2/ARE signaling pathway	Its downregulation attenuated oxidative stress and enhanced cell survival through upregulation of KIAA0319.	Huang et al., 2020
miR-425-5p	Postmortem brain tissue samples from	HEK293/tau	HSPB8	-	Elevates apoptosis and tau phosphorylation through downregulation of HSPB8	Yuan et al., 2020
miR-425-5p miR-339-5p	Blood samples (for PBMC) from 45 AD patients and 41 age- and gender-matched healthy controls	N2a/APPswe	BACE1	-	Can be implicated in AD pathogenesis through modulating expression of BACE1	Ren et al., 2016
miR-25	30 male Kunming mice	Hippocampal neuronal cells	KLF2	Nrf2 signaling pathway	Represses proliferation of hippocampal neuron cells and induced apoptosis in these cells by targeting KLF2	Duan and Si, 2019
miR-138	-	SH-SY5Y	DEK	-	Increases apoptosis rate in SH-SY5Y cells by targeting DEK and downregulation of its expression	Miao et al., 2020
miR-138q	-	N2a/APP, HEK293/tau	RARA	-	Can be implicated in the pathogenesis of AD through the promotion of tau phosphorylation by targeting RARA	Wang et al., 2015b
miR-149-5p	Plasma samples from 30 AD patients and 30 healthy controls	293/APPsw	KAT8	-	Can be implicated in AD pathology by targeting KAT8 to negatively regulate H4K16ac	Zhou et al., 2020
miR-125b	Cerebral tissues from nine AD patients, eight patients with MCI, and 10 normal individuals	Neuronal cells obtained from human fatal cortical tissues	FOXQ1	-	Promotes phosphorylation of Tau and apoptosis of neuronal cells by targeting FOXQ1	Ma et al., 2017
miR-125b	CSF samples from 24 AD patients and 24 healthy controls	Neuro2a APPSwe/Δ9	-	-	Promotes cellular apoptosis, oxidative stress, and expression of inflammatory factors and suppressed cell proliferation by regulating SphK1	Jin et al., 2018
miR-125b	Brain tissue specimens 10 AD patients and 5 healthy controls, C57BL/6 wild-type mice	Primary hippocampal and cortical neuron obtained from embryonic day 19 rat	Bcl-W, DUSP6, PPP1CA	MAPK signaling	Its high expression resulted in increased tau phosphorylation through targeting Bcl-W, DUSP6 and PPP1CA. also its overexpression led to perished associative learning in mice.	Banzhaf-Strathmann et al., 2014
miR-200b miR-200c	Wild-type C57BL/6J mice and Tg2576 mice	PMNCs, SH-SY5Y	-	-	Transfection with miR-200b/miR-200c alleviated memory impairment and improved spatial learning through regulation of S6K1-mediated insulin signaling.	Higaki et al., 2018
miR-200c	Plasma samples from 14 AD patients and 13 normal controls, APPswe/PS1ΔE9 double-transgenic mice	PC12	PTEN	-	Its overexpression improved neuronal survival and neurite outgrowth by targeting PTEN.	Wu et al., 2016
miR-10a	50 male Sprague-Dawley (SD) rats	-	BDNF	BDNF-TrkB signaling pathway	Promotes apoptosis and cell growth arrest by targeting BDNF and inhibition of BDNF-TrkB signaling pathway	Wu et al., 2018b
miR-1908	Blood samples from 20 AD patients and 20 age-matched control individuals	THP-1, U87	ApoE	-	Disrupts clearance of Aβ by ApoE through downregulation of its expression	Wang et al., 2018
miR-139	SAMR1 and SAMP8 mice	Primary hippocampal cell	CB2	-	Its overexpression improved memory function and learning ability by targeting CB2.	Tang et al., 2017
miR-146a	-	SH-SY5Y	Lrp2	Akt signaling pathway	Raised the rate of cellular apoptosis through targeting Lrp2 and inhibition of Akt signaling pathway	Wang et al., 2016
miR-146a	Brain tissues from 17 AD patients, 5xFAD mice	SH-SY5Y	ROCK1	ROCK1/PTEN pathway	Its inhibition decreased phosphorylation of tau proteins and improved memory function by modulating ROCK1 expression.	Wang et al., 2016
miR-146a	Brain tissues from 36 AD patients and 30 control subjects	Primary human astroglial (HAG) cells, primary HNG	IRAK-1	-	Targets IRAK-1 and downregulated its expression so caused a sustained inflammatory response	Cui et al., 2010
miR-33	APPsw/PSEN1Δ9 (APP/PS1) transgenic mice	N2a, N2a-APPsw, H4-APPsw	ABCA1	-	Downregulates expression of ABCA1 and consequently impaired Aβ clearance	Kim et al., 2015
miR-34c	C57 mice	Primary hippocampal neurons, N2a	VAMP2	-	Its downregulation alleviated learning and memory dysfunction and synaptic impairment through targeting VAMP2.	Hu et al., 2015
miR-26b	APP/PS1 double-transgenic mice	N2a, HEK293	IGF-1	-	Augments production of Aβ by targeting IGF-1 and its inhibition reversed these effects	Liu et al., 2016b
miR-98	APP/PS1 mice	HEK293, N2a	IGF-1	-	Its inhibition suppressed Aβ generation and tau phosphorylation by regulating the expression of IGF-1.	Hu et al., 2013
miR-206	Blood samples from 30 AD patients and 30 healthy controls	BV-2	IGF-1	-	Elevates inflammatory responses induced by LPS and promoted the release of Aβ from microglia cell through targeting IGF-1	Xing et al., 2016
miR-574	APP/PS1 double transgenic mice and wild type mice	Primary hippocampal neurons obtained from mice	Nrn1	-	Is involved in the regulation of synaptic activity and cognitive function through targeting Nrn1	Li et al., 2015
miR-26b	Postmortem brain tissues from 10 patients with MCI, 10 patients with AD, and eight controls	Primary cortical neurons obtained from Sprague Dawley rat	Rb1	-	Promotes tau phosphorylation and cell cycle entry and consequently lead apoptosis by targeting Rb1	Absalon et al., 2013
miR-922	-	SH-SY5Y, HEK-293T	UCHL1	-	Enhances phosphorylation of tau proteins by targeting UCHL1 so contributed to AD pathogenesis	Zhao et al., 2014
miR-485-3p	Serum samples from 89 AD patients and 62 healthy controls	SH-SY5Y, BV2	AKT3	-	Its knockdown promoted cell proliferation, inhibited apoptosis and neuroinflammation partly by targeting AKT3. Its expression has been associated with MMSE score, inflammatory response.	Yu et al., 2020

**Figure 1:**
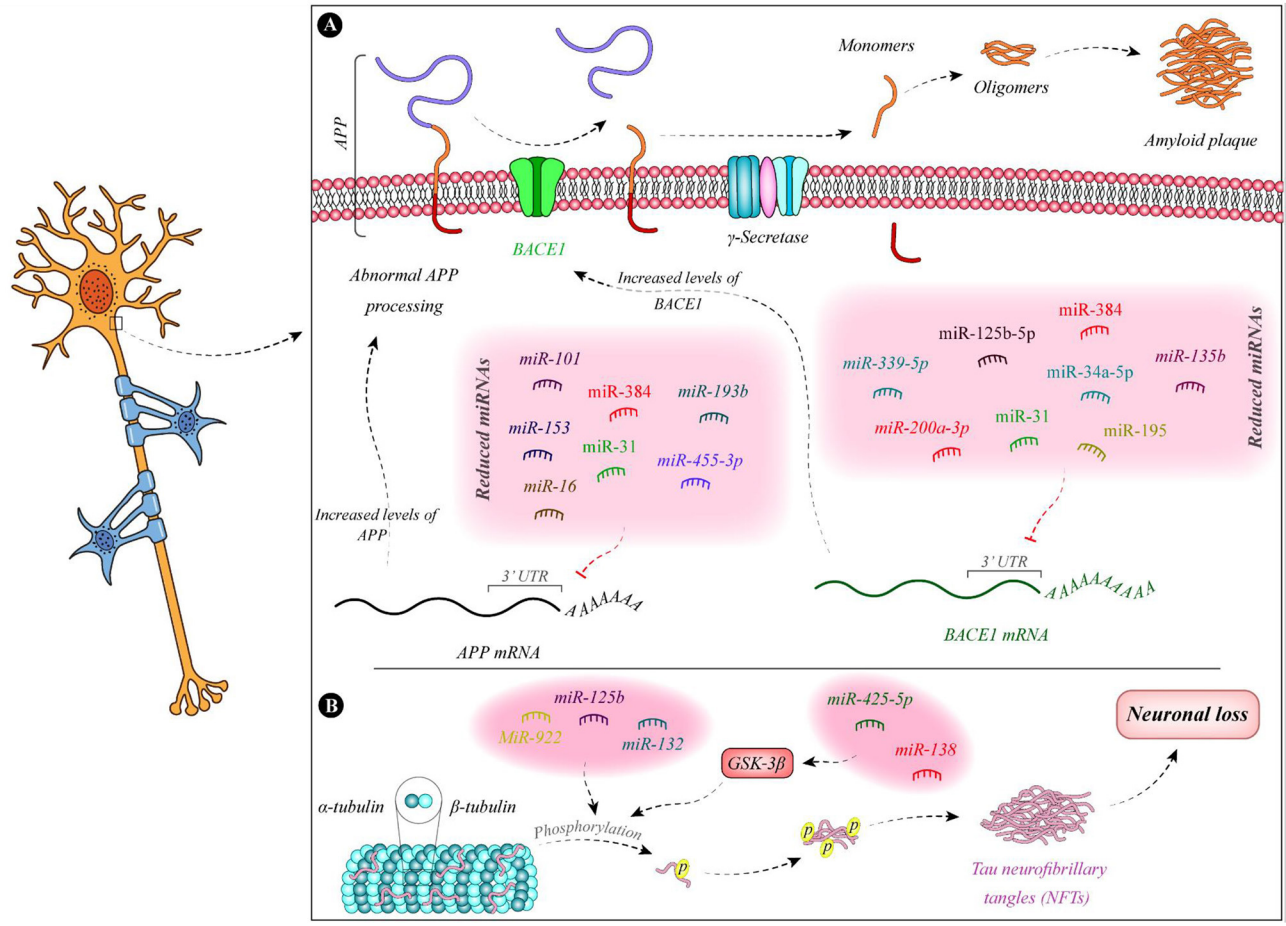
Summary of the function of miRNAs in the pathogenesis of AD. **(A)** Expressions of miR-135b (Zhang et al., 2016b), miR-195 (Zhu et al., 2012), miR-34a-5p (Liang et al., 2020), miR-384 (Liu et al., 2014b), miR-125b-5p (Liang et al., 2020), miR-31 (Barros-Viegas et al., 2020), miR-200a-3p (Pan et al., 2019), and miR-339-5p (Long et al., 2014) are decreased in patients with Alzheimer's disease. These miRNAs bind with the 3' UTR of BACE1 and decrease its expression. Therefore, the downregulation of these miRNAs leads to the upregulation of BACE1. In addition, expression levels of some APP-binding miRNAs namely miR-101 (Vilardo et al., 2010), miR-153 (Liang et al., 2012), miR-16 (Liu et al., 2012), miR-384 (Liu et al., 2014b), miR-31 (Barros-Viegas et al., 2020), miR-193b (Liu et al., 2014a), and miR-455-3p (Kumar et al., 2019) is decreased in patients with Alzheimer's disease. **(B)** Tau phosphorylation leads to defects in microtubules and induction of neurofibrillary tangles which result in neuron death. miR-138 and miR-425-5p are increased in Alzheimer's disease. These miRNAs regulate the expression of GSK-3β and enhance Tau phosphorylation (Wang et al., 2015b; Yuan et al., 2020). In addition, downregulation of miR-132 and upregulation of miR-125b and miR-922 leads to Tau hyperphosphorylation (Zhao et al., 2014; Salta et al., 2016; Ma et al., 2017).

Figure 1 demonstrates the function of a number of miRNAs in the pathogenesis of AD.

## Prognostic and Diagnostic Role of miRNAs in AD

The prognostic role of miRNAs in AD has been assessed in a single study. Xie et al. have evaluated serum levels of miR-206, miR-132, BDNF, and SIRT1 in a cohort of patients with amnestic mild cognitive impairment at baseline and after 5-year follow-up. Their results have shown higher levels of miR-206 in patients who converted to AD both at the baseline and after 5-year follow-up. However, miR-132 levels have been statistically similar between the conversion and non-conversion groups at both time points. Based on the Kaplan-Meier analysis, AD conversion has been correlated with over-expression of miR-206. In addition, multivariate Cox regression analysis has shown the suitability of serum miR-206 and its target BDNF as indicators of AD conversion (Xie et al., 2017). The diagnostic role of several miRNAs has been appraised in AD. For instance, downregulation of miR-433 and miR-133b in serum samples could precisely differentiate between AD subjects and normal persons (Yang et al., 2019b; Wang and Zhang, 2020). Moreover, the expression profile of the former miRNA in the cerebrospinal fluid (CSF) has appropriate diagnostic power for distinguishing AD patients (Wang and Zhang, 2020). The most astonishing results have been obtained for miR-34c. Expression of miR-34c has been increased in both cellular and plasma constituents of blood specimens of AD patients. The area under the receiver operating characteristic curve has been estimated to be 0.99. Moreover, expression of miR-34c has been inversely correlated with mental performance, as described by the mini-mental state examination. miR-34c has also been shown to affect the expression of numerous genes being involved in neuron survival and oxidative processes (Bhatnagar et al., 2014). Expression levels of miR-132 and miR-212 in neural-derived extracellular vesicles have been demonstrated to differentiate patients with AD from healthy subjects, yet their aptitude in identifying both AD and mild cognitive impairment as different from a healthy status has not been suitable (Cha et al., 2019). Table 3 summarizes the outlines of various studies that have reported on the diagnostic value of miRNAs in AD.

**Table 3:** Diagnostic role of miRNAs in AD.

**microRNA**	**Expression pattern**	**Samples**	**ROC curve analysis**			**References**
			**Sensitivity**	**Specificity**	**AUC**	
miR-133b	Downregulated	Serum samples from 105 AD patients and 98 control individuals	90.8%	74.3%	0.907	Yang et al., 2019b
miRNA-101a	Downregulated	Plasma samples from 46 AD patients 60 healthy individuals	0.913	0.733	0.8725	Xiao et al., 2019
miR-433	Downregulated	Serum samples from 118 AD patients and 62 healthy controls	78.8%	80.6%	0.827	Wang and Zhang, 2020
miR-433	Downregulated	CSF samples from 32 AD patients and 12 controls	84.4%	91.7%	0.952	
hsa-miR-21-5p	Downregulated (in AD patients compared with DLB patients)	Plasma extracellular vesicles from 18 patients with dementia with Lewy bodies (DLB), 10 AD patients and 15 age- and sex-matched healthy controls	-	-	0.93	Gámez-Valero et al., 2019
hsa-miR-451a	Downregulated (in AD patients compared with DLB patients)		-	-	0.95	
miR-103	Downregulated (in AD patients compared with PD patients and controls)	Plasma samples from 120 AD patients, 120 patients with Parkinson's disease (PD) and 120 healthy subjects	80.0%	84.2%	0.891	Wang et al., 2020
miR-103	Downregulated (in AD patients compared with PD patients and controls)		86.7%	55.0%	0.775	
miR-107	Downregulated (in AD patients compared with controls)		77.5%	59.2%	0.739	
miR-132	Downregulated	Blood samples (for neurally derived plasma exosomes) from 16 AD patients, 16 patients with mild cognitive impairment (MCI), and 31 controls	-	-	0.58 (distinguishing AD and MCI patients from controls)	Cha et al., 2019
miR-132	Downregulated		-	-	0.77 (distinguishing AD patients from controls)	
miR-212	Downregulated		-	-	0.68 (distinguishing AD and MCI patients from controls)	
miR-212	Downregulated		-	-	0.84 (distinguishing AD patients from controls)	
has-miR-346 has-miR-345-5p has-miR-122-3p has-miR-208b-3p has-miR-1291 hsa-miR-640 has-miR-499a-5p has-miR-650 has-miR-1285-3p has-miR-1299 has-miR-1267 has-miR-206	Upregulated Upregulated Upregulated Downregulated Upregulated Upregulated Downregulated Upregulated Upregulated Upregulated Upregulated Downregulated	Serum samples from 51 controls and 32 AD patients	90.0%	66.7%	-	Zhao et al., 2020
miR-106b	Downregulated	Serum samples from 56 AD patients and 50 healthy volunteers	94%	62%	0.80.	Madadi et al., 2020
miR-16-5p	Downregulated	CSF samples from 17 Young-onset AD (YOAD), 13 Late-onset AD (LOAD) and 12 healthy controls	-	-	0.760	McKeever et al., 2018
miR-451a	Downregulated		-	-	0.951	
miR-605-5p	Downregulated		-	-	0.706	
miR-125b-5p	Upregulated		-	-	0.723	
miR-451a	Downregulated		-	-	0.847	
miR-605-5p	Downregulated		-	-	0.765	
miR-125b-5p	Upregulated		-	-	0.785	
miR-501-3p	Downregulated	Serum samples from 36 patients with AD and 22 age-matched control volunteers	53%	100%,	0.82	Hara et al., 2017
hsa-miR-26a-5p hsa-miR-181c-3p hsa-miR-126-5p hsa-miR-22-3p hsa-miR-148b-5p hsa-miR-106b-3p hsa-miR-6119-5p hsa-miR-1246 hsa-miR-660-5p	Downregulated Downregulated Downregulated Downregulated Downregulated Upregulated Upregulated Upregulated Upregulated	Serum samples 121 patients with AD and 86 healthy controls	-	-	0.987	Guo et al., 2017
hsa-miR-106a-5p	Downregulated	Blood samples from 172 AD patients and 109 healthy controls	68%	93%	-	Yilmaz et al., 2016
miR-31 miR-93 miR-143 miR-146a	Downregulated Downregulated Downregulated Downregulated	Serum samples 79 AD patients and 75 controls	-	-	0.709	Li et al., 2015
miR-342-3p	Downregulated	Serum samples from 208 patients with AD and 205 age- and sex-matched healthy volunteers	81.5%	70.1%	-	Tan et al., 2014
miR-125a-5p	Upregulated	CSF samples from 48 patients with behavioral variant of frontotemporal dementia (bvFTD), 48 patients with AD and 44 healthy controls	74%	82%	0.75	Denk et al., 2018
miR-30a-5p	Upregulated		78%	68%	0.73	
miR-20a-5p	Upregulated	Serum samples from 48 patients with bvFTD, 47 patients with AD, and 38 healthy controls	-	92%	0.85	
miR-29b-3p	Upregulated		93%	-	0.83	
miR-26b-5p	Upregulated		89%	89%	0.97	
miR-320a	Downregulated		83%	90%	0.90	
miR-483-5p	Upregulated	Plasma samples from 20 AD patients, 15 MCI-AD patients and 15 non-demented controls (CTR)	-	-	0.99 (AD vs. CTR)	Nagaraj et al., 2017
miR-483-5p			-	-	0.95 (MCI-AD vs. CTR)	
miR-502-3p	Upregulated		-	-	0.94 (AD vs. CTR)	
miR-502-3p			-	-	0.86 (MCI-AD vs. CTR)	
miR-485-3p	Upregulated	Serum samples from 89 AD patients and 62 healthy controls	84.3%	96.8%	0.933	Yu et al., 2020
miR-425	Upregulated	Blood samples (for PBMC) from 45 AD patients and 41 age- and gender-matched healthy controls	-	-	0.868	Ren et al., 2016
miR-339	Upregulated		-	-	0.761	
miR-206	Upregulated (in aMCI-AD group compared with aMCI-aMCI group)	Serum sample from 458 amnestic mild cognitive impairment (aMCI)	95.5%	77.8%	0.95	Xie et al., 2017
miR-455-3p	Upregulated	Postmortem brain samples from 27 AD patients and 15 controls	-	-	0.792	Kumar and Reddy, 2018
miR-455-3p	Upregulated	Skin fibroblast cell from 4 patients with familial AD, 6 patients with sporadic AD, and eight healthy control	-	-	0.861	
miR-455-3p	Upregulated	Serum samples from 10 AD patients, 20 MCI patients and 18 healthy controls	-	-	0.79	Kumar et al., 2017
miR-455-3p	Upregulated	Postmortem brain tissues from 16 AD patients and 5 controls	-	-	0.86	
miR-34c	Upregulated	Plasma samples from 110 AD patients and 123 control subjects	0.92	0.96	0.99	Bhatnagar et al., 2014
miR-29a	Upregulated	CSF samples from 18 patients with AD and 20 healthy volunteers	89%	70%	0.87	Müller et al., 2016

## miRNA Polymorphisms and Risk of AD

Boscher et al. have screened a larger cohort of early-onset AD (EOAD) patients who did not have autosomal dominant mutations for the presence of genetic polymorphisms. They have recognized 86 copy number variants (CNVs) in miRNA-coding genes, 31 of them being only present in EOAD cases. Duplication of the MIR138-2 locus has been one of these CNVs. Based on the role of miR-138 in Aβ production and tau phosphorylation, this CNV might be implicated in the risk of EOAD (Boscher et al., 2019). Functionally, miR-138 upregulation enhances Aβ synthesis and tau phosphorylation through modulation of GSK-3β and FERMT2 (Boscher et al., 2019). Other studies have demonstrated the role of rs2910164 of pri-miR-146a, rs57095329 of miR-146a, and rs2291418 of miR-1229 precursor in conferring risk of AD (Table 4). Zhang et al. have scanned the coding region of pri-miR-146a in AD patients. Among the four single nucleotide polymorphisms (SNPs) located in this genomic region, rs2910164 has been identified as a risk locus for AD as the C allele of this SNP has enhanced risk of AD.

**Table 4:** miRNA polymorphisms and risk of AD.

**microRNA**	**Polymorphism**	**Samples**	**Population**	**Assay method**	**Function**	**References**
miR-138	Copy number variant (CNV)	Whole exome sequencing data of 546 unrelated patients with early-onset Alzheimer's disease (EOAD) and 597 controls subjects	French	QMPSF	Its duplication was observed in EOAD patients and functional studies showed that miR-138 upregulation caused increased production of Aβ and higher phosphorylation of tau. So miR-138 gene dosage can be a potential risk factor for EOAD.	Boscher et al., 2019
Pri-miR-146a	SNP (rs2910164)	Blood samples from 103 AD patients and 206 healthy controls	Han Chinese	Sequencing	Rare C allele of this SNP was correlated AD and low expression of mature miR-146a-5p.	Zhang et al., 2015
miR-146a	SNP (rs57095329)	Blood samples from 292 AD patients 300 healthy volunteers	Chinese	ABI PRISM SNapShot method	AA genotype of rs57095329 was correlated with an elevated predisposition to AD and was associated with high expression of *miR-146a*.	Cui et al., 2014
miR-1229 precursor	SNP (rs2291418)	Analysis of GWAS data on late-onset AD	-	-	rs2291418 was associated with AD risk. An allele of rs2291418 was correlated with an increased miR-1229-3p expression that targets an AD-related gene, SORL1, so can have an important role in AD.	Ghanbari et al., 2016

Notably, this variant has been shown to reduce the expression of mature miR-146a-5p, releasing TLR2 from its inhibitory effects. Moreover, cell line studies have shown the impact of the C allele on upregulation of expression of TNF-α after induction with β-amyloid. Therefore, this SNP might predispose patients to AD by disturbing the production of mature miRNA and influencing the activity and expression level of TLR2 (Zhang et al., 2015). Cui et al. have analyzed the genotype and allele frequencies of rs2910464 and rs57095329 of miR-146a and have reported that the AA genotype of the former SNP increases susceptibility to AD and results in cognitive reduction in the affected individuals. Contrary to the previously mentioned study by Zhang et al., the risk genotype has been associated with higher levels of miR-146a in the PBMCs of control subjects and has exerted more robust effects on IL-6 and IL-1β synthesis following stimulation with LPS (Cui et al., 2014). Finally, in a genome-wide association study, Ghanbari et al. have detected an association between rs2291418 in the miR-1229 precursor and risk of AD. The risk allele of this SNP has been shown to increase the expression of miR-1229-3p, thus decreasing the expression of SORL1, an AD-associated gene. In addition, among more than 42,000 variants in miRNA-binding regions, 10 variants in the 3' UTR of nine genes have been associated with this disorder; among them has been rs6857, which enhances the miR-320e-mediated modulation of PVRL2 expression (Ghanbari et al., 2016).

## Effects of Herbal/Chemical Agents on the Expression of miRNAs in the Context of AD

Osthole, the active component of the fruits of the genus *Cnidium moonnieri* (L.) *Cussion* has been shown to affect the AD course *via* modulation of miRNAs expression. Lin et al. have shown miR-101a-3p as the main affected miRNA by osthole. APP has been identified as the target of miR-101a-3p. Osthole has enhanced the learning and memory aptitude in an animal model of AD, and it has inhibited APP levels by promoting the expression of miR-101a-3p (Lin et al., 2019). Other studies have verified the effects of Osthole on the expression of miR-9 (Li et al., 2016, 2017). Functionally, osthole enhances the viability of neurons, decreases apoptosis of these cells, and reverses the decline of synaptic proteins in APP-expressing cells by affecting miR-9 expression and consequently decreasing CAMKK2 and p-AMPKα levels (Li et al., 2016). Additionally, osthole has pro-survival effects in APP-expressing neural stem cells through suppression of the Notch pathway (Li et al., 2017). Moreover, Berberine has been shown to enhance proliferation and attenuate neuron apoptosis *via* regulation of miR-188/NOS1 molecular cascade (Chen et al., 2020b). Treatment of Aβ-treated murine microglia and neuroblastoma cells with this substance or upregulation of miR-188 in these cells has accelerated cell proliferation and suppressed caspase-3 activity and apoptosis (Chen et al., 2020b). Finally, exmedetomidine has been demonstrated to accomplish neuroprotective effects and enhance cognitive function in an animal model of AD by modulating the miR-129/YAP1/JAG1 cascade (Sun et al., 2020). Table 5 shows the effects of different AD-modifying compounds on the expression of miRNAs.

**Table 5:** Effect of different compounds on microRNAs.

**microRNA**	**Compound**	**Cell line**	**Animal model**	**Gene/protein interaction**	**Results**	**References**
miR-101a-3p	Osthole	SH-SY5Y	APP/PS1 mice	APP	miR-101a-3p was upregulated by Osthole and its upregulation led to improved memory function and learning capacity and prevented Aβ formation through targeting APP	Lin et al., 2019
miR-9	Osthole	Neural stem cells obtained from newborn C57BL/6 mice	APP/PS1 double transgenic mice	-	miR-9 was upregulated by Osthole and this caused improved cell survival, reduced cell death, alleviated cognitive deficit.	Li et al., 2017
miR-9	Osthole	SH-SY5Y, primary cortical neurons obtained from C57BL/6 mice	-	-	Osthole improved cell survival and suppressed apoptosis through upregulation of miR-9 expression.	Li et al., 2016
miR-34a	Tiaoxin Recipe	-	APPswe/PS1ΔE9 mice	-	Tiaoxin Recipe downregulated expression of miR-34a and ameliorated memory dysfunction, Aβ formation	Boscher et al., 2019
miR-188	Berberine	BV2, N2a	-	NOS1	Berberine enhanced proliferation and inhibited apoptosis partly through regulation of the miR-188/NOS1 axis	Chen et al., 2020b
miR-129	Dexmedetomidine	Primary hippocampal neurons	Male NIH Swiss mice	YAP1	miR-129 was upregulated by Dexmedetomidine and its upregulation led to decreased apoptosis rate and alleviated cognitive decline through targeting YAP1 and prevention of YAP1 interaction with JAG1	Sun et al., 2020

## Discussion

Numerous studies have demonstrated abnormal expression of miRNAs in AD subjects or cell/animal models of AD. However, each miRNA has been the subject of expression and functional assays in few independent studies. miR-146 has been among the miRNAs most assessed in the context of AD, as its expression levels, functions, and polymorphisms have been assessed in association with AD. miR-9 is another AD-associated miRNA whose expression has been altered following treatment of APP-expressing cells with anti-AD substances. In some cases, altered expression of a certain miRNA is regarded as a part of a self-protective process. For instance, the reduction of miR-409-5p expression in the early stages of AD might be associated with lower Aβ-induced synaptic injury (Guo et al., 2019). Similarly, upregulation of miR-200b and miR-200c has protective effects against AD-associated neurotoxicity (Higaki et al., 2018). However, in most cases, an aberrant miRNA signature directly participates in the pathogenesis of AD. miRNAs partake in the pathobiology of AD through various mechanisms, including the regulation of BACE1 activity. miR-200a-3p, miR-195, miR-338-5p, miR-34a-5p, miR-125b-5p, miR-132, miR-384, miR-339-5p, miR-135b, miR-425-5p, and miR-339-5p are among the miRNAs whose interaction with BACE1 has been verified in different investigation. Other miRNAs, such as miR-129-5p, miR-22, and miR-206, might affect the inflammatory responses in the course of AD. Moreover, a number of miRNAs, such as miR-326, miR-338-5p, miR-124-3p, miR-34a, miR-326, and miR-98, modulate apoptotic pathways in neurons, thereby affecting the AD course *via* this route. Tau phosphorylation can be modulated by some miRNAs, namely, miR-200a-3p, miR-326, miR-124-3p, miR-146a, miR-425-5p, and miR-132. Expression of miR-132 has been assessed by several investigations with most of them reporting its downregulation in AD (Wong et al., 2013; El Fatimy et al., 2018; Cha et al., 2019; Deng et al., 2020). Yet, Liu et al. have reported over-expression of miR-132 in patients with mild cognitive impairment and AD vs. normal individuals (Liu and Zhang, 2019).

Abnormal levels of miRNAs in serum or CSF samples have been shown to distinguish AD subjects from normal subjects, indicating their suitability as disease biomarkers. However, these studies have not been validated in independent cohorts. miR-103, miR-126, miR-93, miR-29, miR-424, and miR-181 are among AD-associated miRNAs with biomarker potential whose application as disease biomarkers has been validated in other disorders (So et al., 2020).

Animal studies have shown promising results regarding the impact of miRNA modifications on the course of AD. However, based on the unavailability of brain tissue for therapeutic interventions in human subjects, identification of appropriate transport mechanisms for delivery of anta-/ago-miRs to this tissue is an important issue.

The anti-AD effects of Osthole, Tiaoxin Recipe, Berberine, and Dexmedetomidine have been shown to be exerted through modulation of a number miRNAs, such as miR-101a-3p, miR-9, miR-34a, miR-188, and miR-129, emphasizing further the impact of miRNAs in the progression of AD. However, these results should be verified in human subjects as well.

Few studies have shown the association between miRNA CNVs/ SNPs and the risk of AD. However, these results have not been verified in different ethnic groups. Re-assessment of the results of genome-wide association studies with a focus on non-coding regions might lead to the identification of further risk loci for this multifactorial condition.

Finally, a limitation of several functional investigations in this field is that they have assessed miRNA functions in cell lines such as HEK293 and SH-SY5Y, which are not true models of AD.

## Author Contributions

MT and SG-F wrote the draft and revised it. MS, MH, and MG collected the data, designed the tables, and figures. All authors contributed to the article approved the submitted version.

## Conflict of Interest

The authors declare that the research was conducted in the absence of any commercial or financial relationships that could be construed as a potential conflict of interest.

## Abbreviations

AD: Alzheimer's disease
microRNA: miRNA
Aβ: β-amyloid peptides
BACE1: β-secretase
EOAD: early-onset Alzheimer's disease
CNV: copy number variant
SNP: single nucleotide polymorphism
AChE: Acetylcholinesterase
iNOS: Inducible nitric oxide synthase
ROS: reactive oxygen species
MDA: Malondialdehyde
MAPK: mitogen-activated protein kinase
SOD: Superoxide dismutase
GSH-Px: glutathione peroxidase.
